# Self vs. other, child vs. adult. An experimental comparison of valuation perspectives for valuation of EQ-5D-Y-3L health states

**DOI:** 10.1007/s10198-021-01377-y

**Published:** 2021-10-06

**Authors:** S. A. Lipman, V. T. Reckers-Droog, M. Karimi, M. Jakubczyk, A. E. Attema

**Affiliations:** 1 Erasmus School of Health Policy and Management, Erasmus University Rotterdam, Rotterdam, The Netherlands; 2 Pharmerit, Rotterdam, The Netherlands; 3 SGH Warsaw School of Economics, Decision Analysis and Support Unit, Warsaw, Poland

**Keywords:** Health state valuation, Perspective, EQ-5D-Y, Time trade-off, Child health, I10

## Abstract

**Objectives:**

EQ-5D-Y-3L health states are valued by adults taking the perspective of a 10-year-old child. Compared to valuation of adult EQ-5D instruments, this entails two changes to the perspective: (i) child health states are valued instead of adult health states and: (ii) health states are valued for someone else instead of for oneself. Although earlier work has shown that these combined changes yield different values for child and adult health states that are otherwise equal, it currently remains unclear why. Hence, we aimed to disentangle the effects of both changes.

**Methods:**

A sample of 205 students (mean age: 19.48) was surveyed. Each respondent completed visual analogue scale (VAS) and time trade-off (TTO) tasks for five EQ-5D-Y-3L states, using four randomly ordered perspectives: (i) self-adult (themselves), (ii) other-adult (someone their age), (iii) self-child (themselves as a 10-year-old), (iv) other-child (a child of 10 years old). We compared how each perspective impacted outcomes, precision and quality of EQ-5D-Y-3L valuation.

**Results:**

Overall, differences between perspectives were consistent, with their direction being dependent on the health states and respondents. For VAS, the effect on outcomes of valuation depended on severity, but variance was higher in valuation with child perspectives. For TTO, we observed that EQ-5D-Y-3L states valued on behalf of others (i.e., children or adults) received higher valuations, but lower variances.

**Conclusion:**

The use of a different perspective appears to yield systematic differences in EQ-5D-Y-3L valuation, with considerable heterogeneity between health states and respondents. This may explain mixed findings in earlier work.

**Supplementary Information:**

The online version contains supplementary material available at 10.1007/s10198-021-01377-y.

## Introduction

The growing interest in the use of cost-utility analyses for paediatric populations [22] necessitates measurement and valuation of health-related quality of life (HRQOL) in children and adolescents. Valuation of child and adolescent health, however, is associated with several methodological challenges, specifically associated with the methods, sample and perspective used for valuation [20, 26, 39, 46]. Although various measures of HRQOL for adult and paediatric populations exist [7], decision bodies in several countries recommend using EQ-5D instruments for measurement and valuation of HRQOL (e.g., [18, 30, 53]. For assessing HRQOL in children aged 8 to 15 years, the EQ-5D-Y-3L is recommended [14] and a protocol to obtain value sets for estimation of quality-adjusted life years (QALYs) in health technology assessment has been released recently [35]. Similar to obtaining value sets for adult EQ-5D instruments (i.e., EQ-5D-3L and -5L), EQ-5D-Y-3L value sets are following protocol obtained using a sample representative of the general adult population. However, whereas for adult instruments respondents value EQ-5D health states for themselves [8, 12], EQ-5D-Y-3L health states are valued by adults using the perspective of a 10-year-old child^1^ [35].

Several authors have demonstrated that the perspective used influences valuation of EQ-5D health states [11, 19, 21, 28, 40]. However, it remains unclear what drives these differences, which may (at least in part) be explained by the fact that the prescribed perspective for valuation of EQ-5D-Y-3L comprises not one but two primary changes from the perspective prescribed for health state valuation of adult EQ-5D instruments [34]. First, instead of adults valuing health states for their adult selves, they value health states for a child. This entails a change from an adult to a child perspective, which we denote as Δ(A–C). Second, instead of adults valuing health states for themselves, they value health states for someone else. This, in turn, entails a change from decision-making for the self to the other, which we denote as Δ(S–O). It has previously been hypothesized that both changes could separately influence decision-making in valuation tasks [26]. Disentangling the effect of these changes in perspective may require a factorial decomposition that combines Δ(A–C) and Δ(S–O), and hence provides an exploration of all possible combinations of the two primary changes. This decomposition yields four perspectives: self-adult (SA), other-adult (OA), self-child (SC), and other-child (OC).

Previous empirical research into valuation of EQ-5D-Y-3L has used only a subset of these four perspectives simultaneously for examining the impact of the perspective on EQ-5D-Y-3L valuation (see Table 1 for an overview of the extant literature). Most of these studies appear to have selected methods and perspectives based on pragmatism and consistency with the methods used for adult EQ-5D valuation [35]. As a result, the studies presented in Table 1 predominantly used time trade-off (TTO) and discrete choice experiments (DCE), the preferred methods for valuation of EQ-5D [41]. All studies used the prescribed OC perspective for obtaining values for EQ-5D-Y-3L health states, some in combination with the SA perspective that is prescribed for valuation of adult EQ-5D health states [41]. Kreimeier et al. [21] find some evidence for higher valuation for the OC perspective compared to SA, using TTO and DCE. Shah et al. [40] reach similar conclusions for the worst possible health states described by EQ-5D-Y-3L, with the same methods. The latter also hold for visual analogue scales (VAS) and the location of dead (LOD) method [9]. The study designs applied by Kreimeier et al. [21] and Shah et al. [40], however, do not allow any conclusions on the relative contribution of Δ(S–O) and Δ(A–C) to (differences in) EQ-5D-Y-3L valuations. The only study that further decomposed the impact of Δ(S–O) and Δ(A–C) on valuations is that by Kind et al. [19], who used the SA, OA, and OC perspectives. Their results, however, are in contrast to those of others as Kind et al. [19] found evidence for lower valuation of child health states valued with VAS, which is an effect in the opposite direction to that observed by Kreimeier et al. [21] for TTO and by Shah et al. [40] for TTO *and* VAS. It could therefore be hypothesized that differences between TTO and VAS in the impact of the perspective may be explained by differences in willingness to trade-off life duration between perspectives, but this has not yet been explored.

**Table 1:** Empirical evidence on the impact of (different) perspectives on EQ-5D-Y-3L health state valuations

Authors	Methods	Perspectives			Sample(s) and design	Differences	Attributable to
			Δ(A–C)				
Kind et al. [19]	VAS	Δ(S–O)	OA	OC	Adults: SA and OC or SA and OA	OA > SA > OC	Δ(S–O) and Δ(A–C) or order effects
			SA				
			Δ(A–C)				
Kreimeier et al. [21]	TTO and DCE	Δ(S–O)		OC	Adults: OC and SA	TTO: OC > SA (for 3L) DCE: OC ≠ SA	Δ(S–O) and/or Δ(A–C)
			SA				
Mott et al. [28]	DCE	Δ(S–O)		OC	Adults: OC Adolescents: SC	DCE: OC ≠ SC	Δ(S–O) or sample composition
				SC			
			Δ(A–C)				
Shah et al. [40]	TTO and DCE and VAS and LOD	Δ(S–O)		OC	Adults: OC and SA	All methods: OC > SA	Δ(S–O) and/or Δ(A–C)
			SA				
			Δ(A–C)				
Dewilde et al. [11]	TTO and VAS	Δ(S–O)	OA	OC	Adults: OA and OC	TTO: OC > OA VAS: OC > OA	Δ(A–C)

In this study, we advance earlier work on the impact of the change in perspective on EQ-5D-Y-3L valuation, by conducting a within-subjects experiment in which respondents completed TTO and VAS tasks from all four perspectives. Our study, thus, aims to provide additional insight into the way in which EQ-5D-Y-3L valuation depends on the perspective used. To facilitate a within-subjects design, we explored the impact of the four perspectives on EQ-5D-Y-3L valuation using TTO and VAS tasks, rather than the prescribed TTO and DCE tasks [35]. Furthermore, the use of VAS tasks enabled us to obtain further insight into the so far conflicting results for VAS found in the literature [19, 40].

The main objectives of this study were to explore (i) the extent to which the outcomes, precision, and quality of EQ-5D-Y valuation differ between the SA, OA, SC, and OC perspectives, (ii) what change(s) in perspective may drive any observed differences, and (iii) what influence the type of valuation task may have on any observed differences. Obtaining insight into the underlying mechanism driving differences in EQ-5D-Y-3L valuation may be of empirical interest and aid researchers in interpreting the results of valuation studies. Our results may also inform discussions on whether the change in prescribed perspective for valuation of EQ-5D-Y-3L health states—and the implications this change may have for estimation of QALY gains in health technology assessment—is considered desirable [26].

## Methods

To meet the objective of our study, we designed a within-subjects experiment that was conducted using a sample of 205 bachelor students in Business Administration in September and October, 2020. The sample size was informed by a-priori power calculations, with the aim of at least being able to detect differences that classify as ‘small effects’ in a pairwise comparison between perspectives^2^. Respondents were rewarded course credits for their participation, which lasted for 30 min. The sample consisted of 106 females (51.7%) and mean (SD) age was 19.48 (2.33). Respondents completed the experiment in individual cubicles, with an experienced researcher (SL or VRD) present to answer questions. The experiment was programmed in Shiny R (code available upon request).

### Experimental procedure and design

The first part of the experiment was modelled in accordance with the EQ-5D-Y-3L valuation protocol [35]. As such, the experiment started with respondents completing the EQ-5D-Y-3L instrument to familiarize themselves with its descriptive system, as well as reporting their age and sex. Next, the experiment commenced with a pre-recorded instruction video (see Online Supplements) in which the applied valuation tasks were explained by one of the researchers (SL)^3^. The respondents then valued EQ-5D-Y-3L health states across the severity range (see section “Health states” below) using the four perspectives: SA, OA, SC, and OC. We presented the health states and perspectives in random order to respondents. Respondents first valued the health states with VAS and subsequently with TTO (see section “Health state valuation” below). We did not randomize the order of VAS and TTO tasks to account for a possible learning effect that could affect the valuation of each health state, as TTO is often considered more difficult than VAS [5].

### Health states

Health states were drawn from EQ-5D-Y-3L, which describes HRQOL using five dimensions: (i) mobility, (ii) looking after myself, (iii) usual activities, (iv) pain or discomfort and (v) feeling worried, sad, or unhappy. Problems on each dimension are described by three levels: having no problems (level 1), having some problems (level 2), or having a lot of problems (level 3). As such, the worst health state described by EQ-5D-Y-3L is, for example, coded as 33333. To examine the impact of the four perspectives across the severity range, we selected two blocks of health states from Kreimeier et al. [21] with the objective to include one mild, one moderate, and one severe health state. Furthermore, in both blocks we included 22222 and 33333 to enable testing for violations of logical consistency. Block 1 comprised the following health states: 11121, 22222, 32211, 33323, and 33333. Block 2 comprised: 11112, 11312, 22222, 13331, and 33333. Respondents were randomly assigned to one of these blocks.

### Valuation methods

The written TTO and VAS task instruction for the four perspectives can be found in Table 2. VAS was operationalised by asking respondents to value health states using a slider that ranged from 0 to 100, with 0 representing ‘worst imaginable health’ and 100 representing ‘best imaginable health’. TTO was operationalised by a composite TTO procedure [17]. As such, each task introduced a life in impaired health, described as 10 years in a selected EQ-5D-Y-3L health state (Q_i_), followed by immediate death. Valuation commenced with a choice between 10 years in Q_i_ and immediate death. If respondents preferred living 10 years in Q_i_ over immediate death, valuation continued with a choice between 10 years in Q_i_ and 5 years in full health. By means of a bisection elicitation procedure with five choices in total, we obtained indifferences of the form: 10 years in Q_i_ ~ X years in full health (with ~ denoting indifference). Under the assumption of a linear QALY model, such an indifference yielded the utility of health state Q_i_ as X/10 (Torrance [42]). If, however, respondents preferred immediate death over living 10 years in Q_i_, an additional valuation task was required for valuation of this ‘worse-than-dead’ health state [10]. Valuation then continued with the choice between 5 years in full health and 10 years in full health followed by 10 years in Q_i_. We then used the same bisection elicitation procedure to elicit respondent’s indifferences of the form: X years in full health ~ 10 years in full health followed by 10 years in Q_i_. This yielded the utility of health state Q_i_ as (X−10)/10 [4, 10].

**Table 2:** TTO and VAS task instruction for each of four perspectives

	Self	Other
Child	Self-Child (SC)	Other-Child (OC)
	VAS: please rate the following health state for yourself as a 10-year-old child TTO: ‘Which life would be better for yourself as a 10-year-old child?’	VAS: please rate the following health state for a 10-year-old child TTO: ‘Which life is better for a 10-year-old child?’
Adult	Self-Adult (SA)	Other-Adult (OA)
	VAS: please rate the following health state for yourself TTO: ‘Which life is better for yourself?’	VAS: please rate the following health state for someone else (the same age as you) TTO: ‘Which life is better for someone else (the same age as you)?’

### Statistical analyses and modelling strategy

We examined differences in valuation between the perspectives on three distinct levels, i.e., the outcomes, precision, and quality of the valuations. Note that we report the results without correction for multiple hypothesis testing considering that the objective of our study is of exploratory nature.

#### Valuation outcomes

The impact of the different perspectives on the outcomes of EQ-5D-Y-3L valuations was first explored by analysing within-subjects response patterns. These patterns illustrate the degree to which individual respondents valued the same state differently between the four perspectives, with a maximum of four unique valuations per health state (one for each perspective). Furthermore, the direction of the impact of deciding for children and for others was explored by calculating individual-level differences between valuations from each perspective. Next, the overall impact of the different perspectives was investigated using linear mixed-effects regression models for all health states combined. Models were specified with subject random effects with the following fixed effects: (1) Δ(A–C), i.e., a dummy that distinguishes between the two adult (SA and OA) and child (SC and OC) perspectives, (2) Δ(S–O), i.e., a dummy that distinguishes between the two self (SA and SC) and other (OA and OC) perspectives, and (3) Δ(A–C) × Δ(S–O), i.e., an interaction term between these dummies that distinguishes the effect of deciding for another child (i.e., OC). Note that for these analyses we compiled the observations per valuation method and controlled for health state severity in one of two ways, resulting in four models. Models 1 and 2 report the results when controlling for health state severity by health state dummies (reference health state 11112), whereas models 3 and 4 report the results when controlling for health state severity by their level-sum-score (LSS), which is the sum of all problem level values for the five dimensions. That is, the reference health state 11112 has an LSS of 6 and 33333 has an LSS of 15. For ease of interpretation, we ran the regressions with LSS^r^, i.e., LSS^r^ = LSS $-6$. The latter models also allow testing of interactions between health state severity and Δ(A–C) or Δ(S–O).

#### Valuation precision

The impact of the different perspectives on the precision of valuations was explored by comparing variances between perspectives using a Bayesian modelling approach, operationalized as a JAGS model run in R. We opted for a Bayesian estimation process with non-informative priors, as this offers a more flexible and intuitive comparison of variances that can take into account all observations of our within-subjects experiment simultaneously. Below, we present the general approach that was based on Golicki et al. [15], while the formal specification is available as Online Supplement. As Golicki et al. [15], we used a random-parameters model, which assumes that average (dis)utility associated with health states can differ between respondents. To estimate differences in standard deviation (SD) between perspectives we made the additional assumption that variances can differ between health states, as they are generally larger for more severe states [21, 49]. The impact of the perspectives on variance is modelled by two variance scaling factors (VSF) that capture Δ(A–C) and Δ(S–O). The VSF identifies differences in variance between adult and child perspectives as VSF-AC, with the VSF for Δ(S–O) denoted as VSF-SO. We used a Markov Chain Monte Carlo simulation with 500, 4000, and 20,000 adaptive, burn-in, and actual iterations, respectively, without thinning, and two chains. Besides reporting the median point estimate for the posterior distributions of VSF-AC and VSF-SO for VAS and TTO, we report the 2.5th and 97.5th percentiles to construct 95% credible intervals (CrI). We interpret a CrI that does not contain 1 as evidence for a difference in variance between perspectives. If the CrI for a scaling factor falls completely below (above) 1, this indicates that variances are smaller (larger).

#### Valuation quality

The impact of the different perspectives on the quality of valuations was explored following the approach used by Alava et al. [1]. As such, we categorised the following responses as problematic: (i) violations of dominance (e.g., assigning state 11121 a higher value than 22222), (ii) overall non-discrimination (i.e., assigning the same value to all health states within a perspective), and (iii) non-attendance (i.e., exiting the valuation task at the earliest point possible, which was 50 for VAS and − 0.5/0.5 for TTO. Furthermore, we compared how the occurrence of three additional problems related to the VAS and TTO methods differed between perspectives. Specifically, we compared end-point usage for VAS (i.e., the use of 0 and 100 scores) as earlier work has identified that VAS may suffer from end-point aversion [43]. Finally, we compared the occurrence of non-trading responses (i.e., utilities of 1.0) and all-in trading (utilities of − 1.0) for TTO, which may yield TTO data of problematic quality and/or lead to exclusion in other valuation studies due to outlying preferences [4, 13].

## Results

Information about the means (and variance of) and distribution of VAS scores and TTO utilities per state and perspective can be found in the Online Supplements.

### Valuation outcomes

#### Within subjects-effects

**Fig. 1:**
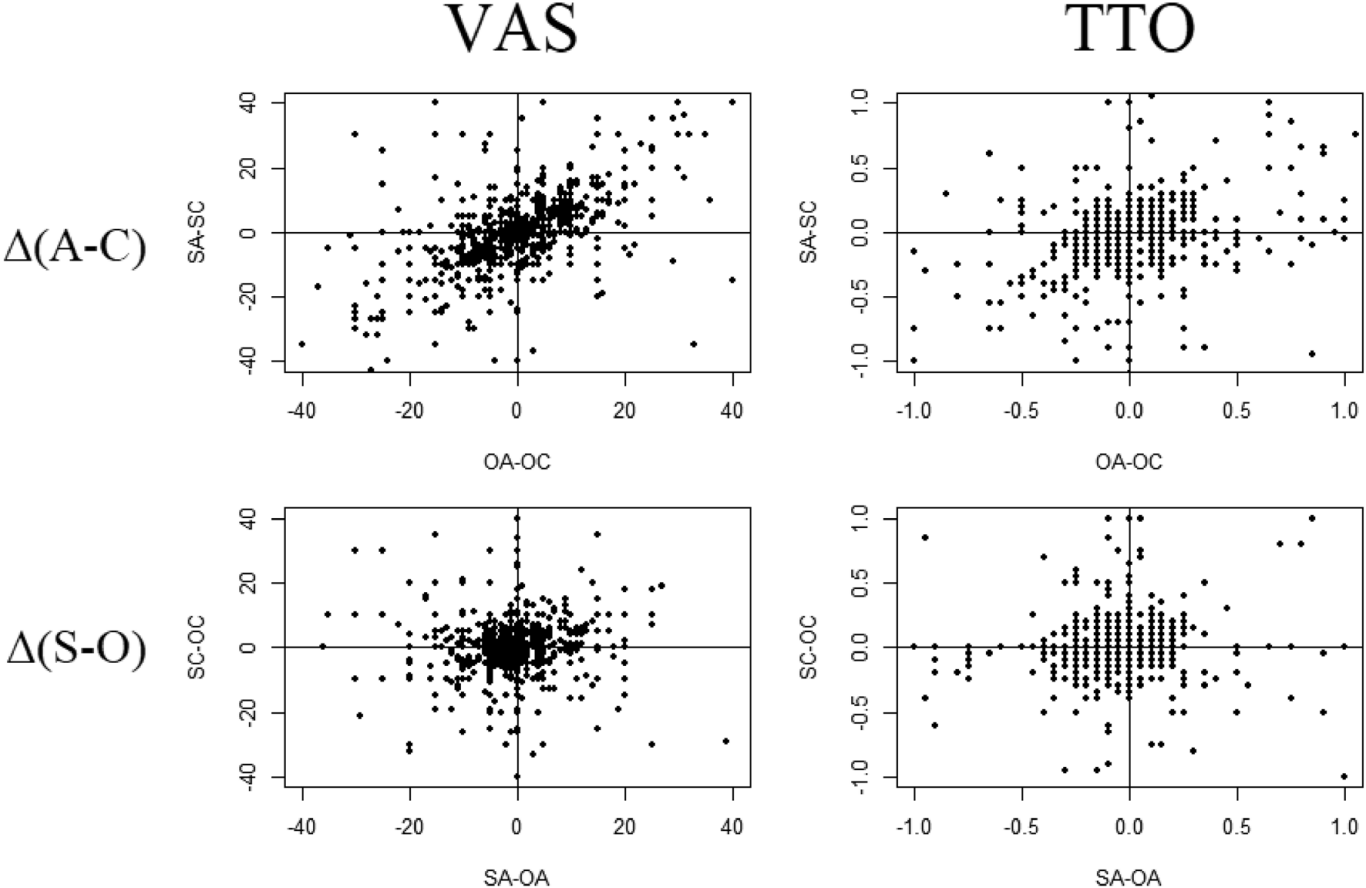
Within-subjects differences between perspectives for VAS (left) and TTO (right). Upper panels show differences between child and adult perspectives (Δ(A–C)) and lower panels show differences between self and other perspectives Δ(S–O)

The observed within-subjects valuations differed substantially between perspectives. For example, we found that for VAS, the mean (SD) number of unique scores per respondent was 2.99 (0.94) out of four possible unique valuations per health state. The direction of the change varied between respondents. In some cases, these changes resulted in health states being considered better-than-dead when valued from one perspective but worse-than-dead from another. This, for example, occurred in 19% of respondents for state 33333. We further explored the impact of perspectives by determining the within-subjects effect of deciding for children and deciding for others. The following strategy was used for deciding for children: we calculated two difference scores per respondent per health state, i.e., (SA–SC) and (OA–OC). These difference scores may reflect the effect of deciding for children instead of adults within both self or both other perspectives respectively. A similar strategy may be used to investigate the effect of Δ(S–O) within subjects, i.e., by calculating (SA–OA) and (SC–OC). Figure 1 shows these difference scores, and indicates large heterogeneity between respondents completing VAS and TTO tasks for children rather than adults. That is, many respondents valued health states higher for children than for adults, while the opposite was also observed (see Online Supplements for a classification per state). Overall, the difference scores were moderately correlated for both methods [VAS: *r*(1018) = 0.51, *p* < 0.001; TTO: *r*(1018) = 0.38, *p* < 0.001], indicating that the observed heterogeneity was systematic (correlations per health state can be found in Online Supplements). This systematicity reflects that although the direction of the impact of deciding for children differed between respondents, it was often consistent within-subjects. In other words, if a respondent valued a health state higher for a child than an adult in both self-perspectives (SA-SC < 0), the same state was most likely also valued higher for a child in both other-perspectives (OA–OC < 0). If the within-subjects effect of deciding for children was in the opposite direction, this consistency held (i.e., SA–SC > 0 and OA–OC > 0). With regard to deciding for others, we observed a weak correlation between differences scores for TTO (*r*(1018) = 0.13, *p* < 0.001), but not for VAS (*r*(1018) = 0.00).

#### Overall effects

**Fig. 2:**
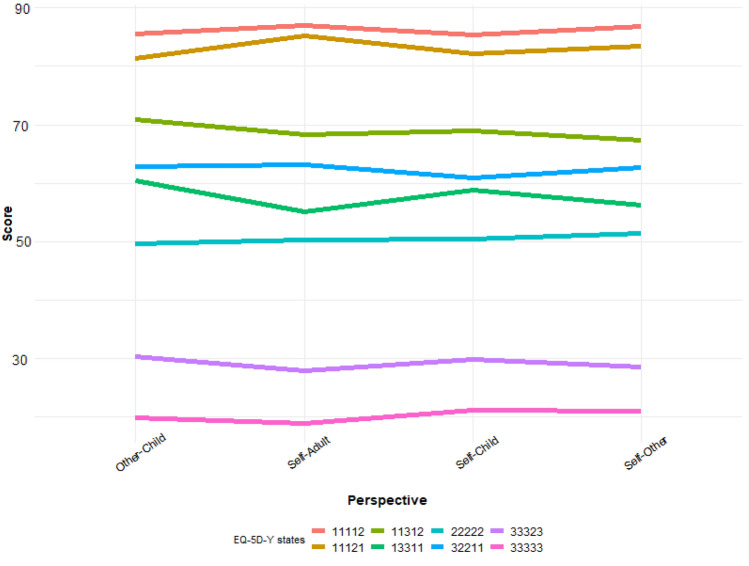
Mean VAS scores per EQ-5D-Y health state and perspective

**Fig. 3:**
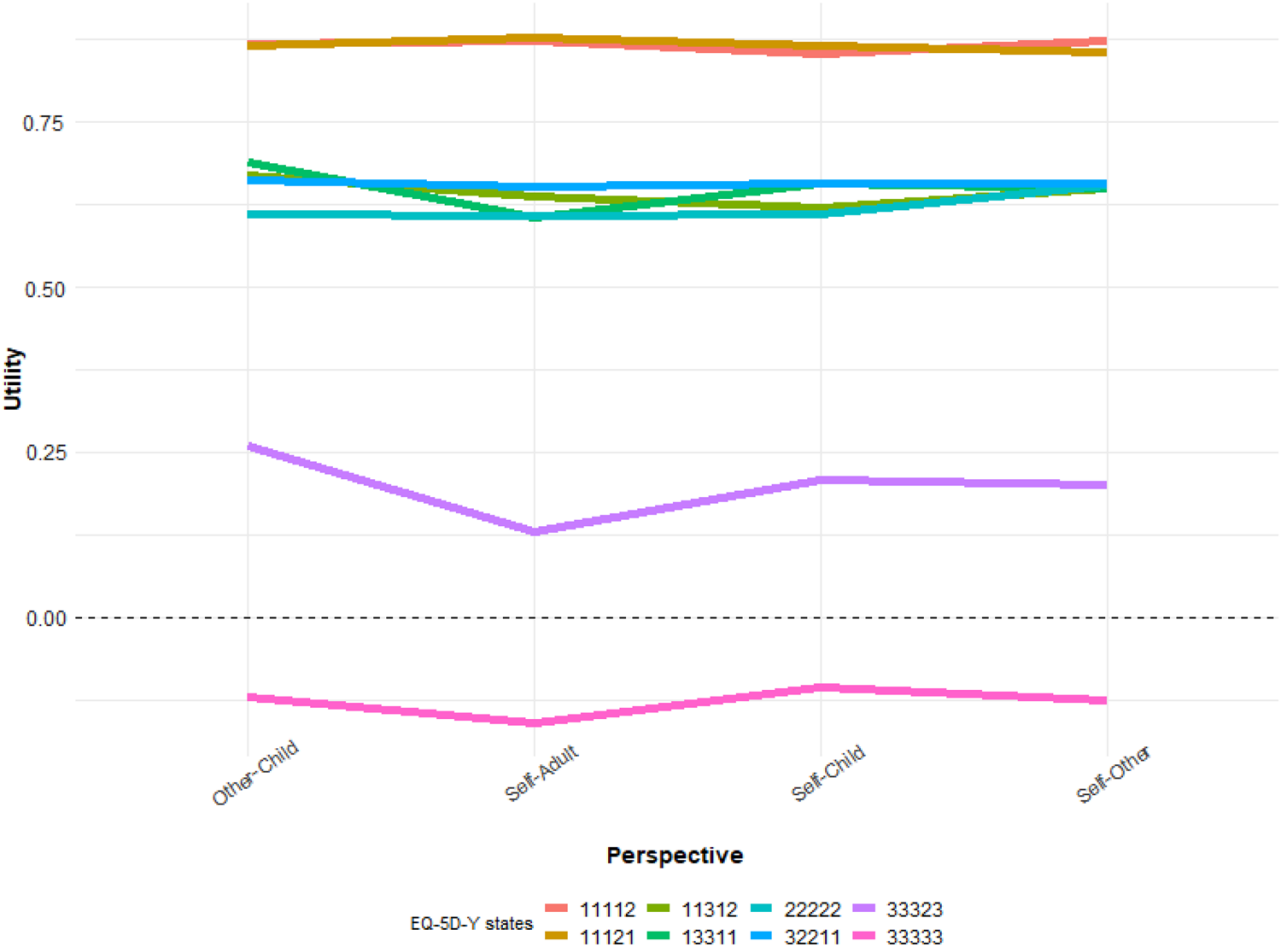
Mean TTO utilities per EQ-5D-Y health state and perspective

Figures 2 and 3 show the impact of perspective on valuation outcomes for VAS and TTO, respectively. Although our within-subjects analyses suggest that considerable heterogeneity exists, overall, the differences between perspectives are generally small to non-existent between health states. In those cases where we did observe (larger) differences, their directions depended on the health state. This is substantiated by a set of regression analyses per health state, reported in the Online Supplements. The results of the linear mixed effects regressions, reported in Table 3, show that, controlling for health states, deciding for others yielded higher TTO utilities (compared to deciding for oneself). However, this was not observed when controlling for LSS (Models 3 and 4). Instead, a positive interaction between LSS and Δ(A–C) was observed, which suggests that although severe health states receive lower valuations, this effect may be less pronounced for children than for adults.

**Table 3:** Fixed effects estimates (standard errors in brackets) for mixed effects regression analyses for VAS scores and TTO utilities

	Severity approach 1: health state dummies		Severity approach 2: LSS	
Model	1	2	3	4
Method	VAS	TTO	VAS	TTO
Intercept	85.74 (1.34)***	0.84 (0.02)***	82.86 (1.28)***	0.94 (0.02)***
Fixed effects				
Δ(A–C): C	0.47 (0.75)	0.02 (0.01)	− 1.24 (1.23)	− 0.02 (0.02)
Δ(S–O): O	0.46 (0.75)	0.03 (0.01)*	− 0.72 (1.23)	0.01 (0.02)
Δ(S–O) × Δ(A–C): OC	− 0.32 (1.05)	− 0.02 (0.02)	1.56 (1.74)	0.01 (0.03)
LSS^r^			− 7.25 (0.17)***	− 0.11 (0.003)
LSS^r^ × Δ(A–C): C			0.42 (0.24) +	0.01 (0.004)*
LSS^r^ × Δ(S–O): O			0.29 (0.24)	0.00 (0.00)
LSS^r^ × Δ(S–O) × Δ(A–C): OC			− 0.46 (0.33)	− 0.01 (0.01)
HS: 11121	− 2.95 (1.42)*	0.00 (0.03)		
HS: 11312	− 17.33 (1.10)***	− 0.22 (0.02)***		
HS: 13311	− 28.54 (1.10)***	− 0.22 (0.02)***		
HS: 22222	− 35.70 (1.03)***	− 0.25 (0.03)***		
HS: 32211	− 23.63 (1.42)***	− 0.21 (0.03)***		
HS: 33323	− 56.88 (1.42)***	− 0.67 (0.03)***		
HS: 33333	− 65.92 (1.03)***	− 0.99 (0.02)***		

LSS^r^ = rescaled level-sum-score; ***, **, * and + indicate (marginal) significance at *p* < 0.001, *p* < 0.01, *p* < 0.05 and *p* < 0.10, respectively

### Valuation precision

Table 4 shows the results of the Bayesian approach used to estimate differences in variance. For VAS, the posterior distribution for VSF-AC suggested that variances were larger for adult perspectives than for child perspectives. For TTO, variances were similar between child and adult perspectives, but the variance of TTO utilities was smaller when respondents valued health states for other adults as compared to for themselves.

**Table 4:** Medians and 95% CrI for scaling factors that identify between-subjects differences in variance

Method	Parameter	Median	95% CrI
VAS	VSF-AC	1.053	[1.008, 1.101]
	VSF-SO	0.970	[0.928, 1.013]
TTO	VSF-AC	1.043	[0.995, 1.092]
	VSF-SO	0.902	[0.861, 0.942]

### Valuation quality

Table 5 provides an overview of the quality indicators per perspective. A higher count indicates lower data quality, except for the indicator ‘end-point usage’, where a higher count indicates higher quality data. The proportion of problematic responses ranged from 0 to 15% of responses between categories. Our results suggest that violations of dominance were not independently distributed, yet occurred more frequently for child perspectives (in particular, for OC). Respondents were also more likely to use the end-point of the VAS for children. Finally, non-trading was more likely for respondents deciding for themselves than for children.

**Table 5:** Quality indicators per perspective (with the maximum possible violations *per perspective* in brackets)

Quality indicator	Self-adult	Other-adult	Self-child	Other-child
Dominance violation (max. *n* = 1406)				
VAS*	101	102	129	214
TTO*	79	68	92	208
Overall non-discrimination (max *n* = *205)*				
VAS	0	0	1	0
TTO	0	0	2	0
Non-attendance (max *n* = 1025*)*				
VAS	51	56	46	32
TTO	93	106	95	85
End-point usage (max *n* = 1025*)*				
VAS*	65	45	89	81
Non-trading responses (max *n* = 1025*)*				
TTO*	62	46	79	63
All-in trading responses (max *n* = 1025*)*				
TTO	37	27	33	27

*Indicates that the distributions was not independent between perspectives, Chi-squared tests, *p* < 0.05

## Discussion

In this study, we explored the impact of the perspective on valuation of EQ-5D-Y-3L health states in a sample of students, by comparing the decomposed influence of two primary changes: (i) deciding for an adult or a child, and (ii) deciding for oneself or another person. Overall, the results of our study show that the four decomposed perspective impact the outcomes, precision, and quality of EQ-5D-Y-3L valuation differently. The overall impact appears to be small. Nonetheless, our work provides detailed insight into what drives differences between similar EQ-5D-Y-3L health states for children and adults that can be found in the literature. Our results suggest that the effect of perspective is highly heterogeneous between health states and respondents. That is, deciding for children and deciding for others yielded higher valuations for some health states and respondents, whereas the opposite was also observed. As such, the results of this study may provide an explanation for some of the (sometimes conflicting) findings in other work comparing the use of different perspectives in EQ-5D-Y-3L with VAS and TTO.

Our findings for VAS valuation outcomes suggest that VAS scores may differ systematically when elicited from an adult or child perspective, but that differences could potentially be small and occur in either direction. These results both align and conflict with earlier work. That is, Kind et al. [19] found a near-uniform pattern of lower valuations for children. Other work by Dewilde et al. [11], found that when adults decide for another adult or for a 10-year-old child, VAS scores are generally lower for adults. Similarly, Shah et al. [40] found that state 33333 is valued higher for children. Our work supports this ambiguity, as we find effects in both directions. For TTO, our results on the effect of deciding for children or adults are similarly mixed. Although some authors have found evidence of higher TTO utilities for children than for adults [11, 40], existing work is also not conclusive about the effect of deciding for children rather than for adults. For example, Kreimeier et al. [21] found that differences between SA and the OC perspective occurred in both directions depending on the health state valued, similar to those observed in the current study.

The heterogeneity in valuations for children, observed in both our study and the extant literature, could have different explanations. Dewilde et al. [11], for example, identified conflicting beliefs about the impact of health impairment on adults and children in a think-out-loud study. Some respondents believed adults were better able to cope with any impairment than children, while others believed the opposite to be true. Other conflicting arguments provided were the importance of adult health to be able to take care of children, as well as the importance of childhood as a foundational period for further development. This suggests that the influence may depend on who is valuing the health state. Our results also show a trend suggesting a relationship between the severity of the health state considered and differences between health states valued for adults and children–suggesting that differences depend on and may increase with severity. Hence, differences between studies could be related to the states selected. However, this should be substantiated in future work.

Few studies have compared the effect of deciding for others and deciding for oneself. Our study showed that for VAS this could have effects in either direction. Hence, our results are similar to those of Kind et al. [19], who found evidence for differences in VAS scores between adults deciding for themselves (SA) or others (OA) for a small number of health states, but the direction of these effects differed between countries and health states. For TTO, our results suggest that deciding for others may have a small but upward effect on utilities overall, which to our knowledge is a novel result. This implies that respondents were less willing to give up life years for others. Future work could explore why this occurs. For example, it may be the case that individuals discount others’ life years less (and are thus less willing to give up others’ years in the future). There is some evidence suggesting such differences in time preferences for self and others [2, 32, 38, 52], but these findings typically involve monetary outcomes and are inconclusive about the direction of these differences. Alternatively, some respondents may feel hesitant to give up others’ life years as they feel it is not their choice to make. Earlier work has found effects in a similar direction related to religion and beliefs in life after death [16, 48], which could reflect a similar position held by individuals trading off their own health.

Our study also allowed exploring valuation precision and quality between perspectives. Although such analyses may be relevant in the context of EQ-5D-Y-3L valuation, they have not extensively been reported in earlier work comparing perspectives for valuation of EQ-5D-Y-3L (an exception is: [28]. Our findings suggested that more violations of dominance occurred when health states were valued for children rather than adults, which is analogous to the findings reported for data quality in [28]. Our work is, to our knowledge, the first to report lower variance observed for EQ-5D health states valued for someone else than for oneself in TTO. This suggests that respondent’s decisions about health states are more similar when deciding for others than when they deciding for themselves. A possible explanation can be found in construal level theory [44]. This theory states that psychological distance affects whether individual’s thought processes are concrete or abstract. Hence, for themselves (low psychological distance), individuals are more likely to take into account their own concrete situation but for others (high psychological distance) people focus less on details. However, we also find higher variance for VAS valuation from adult perspectives, which seems to be in conflict with this explanation. Hence, future work may further explore the causes and implications of variance in valuations.

Overall, the observed differences between perspectives may be considered small in comparison to those observed by others [11, 40]. Seeing as in our study the differences observed were smaller by an order of magnitude (e.g., the regression coefficient for Δ(S–O) was 0.03 for TTO), this raises the question whether these differences are meaningful. We do for three reasons. First, it may help to emphasize that a difference of, for example, 0.03 is the result of individuals trading off 3% more of their remaining lifetime in TTO. Even though these are hypothetical questions, we would caution against classifying such a sacrifice as trivial. The fact that many individuals are loss averse and would give up these life years reluctantly underlines non-triviality [6, 24]. Second, the differences observed in our study are in line with many of the estimated minimally important differences for EQ-5D [27, 50], suggesting that differences of this magnitude may at least be of clinical relevance. Third, the observed differences are of a magnitude that could be seen as practically relevant for decision makers, as the median incremental QALY gain in published cost-effectiveness studies was estimated to be of similar size Wisløff et al. [51].

The discrepancies (in direction and magnitude) between our results and those of others may further be related to the following limitations of our study. First, in most of the extant studies, EQ-5D-Y-3L valuation was completed in one-to-one personal interviews facilitated by a trained interviewer [11, 21, 40]. Our study, instead, asked respondents to work through these tasks by themselves after receiving a video instruction. Some evidence exists that individuals completing these tasks without supervision (i.e., online) may yield data of lower quality [31]. Unfortunately, our experimental set-up, as well as restrictions relating to the COVID-19 pandemic, precluded the use of one-to-one personal interviews in the lab or the use of digital interviewer-assisted interviews [23]. Note that we aimed to compensate for this by having an experienced researcher present at all times. However, our analyses of data quality suggest that the data obtained from this study is of reasonable quality. For example, Ramos-Goñi et al. [36] report that even with personal interviews and extensive quality control, 19% (25%) of Dutch (Spanish) respondents had at least one inconsistent response in EQ-5D-5L valuation. Alava et al. [1] investigated the quality of the data reported in Devlin et al. [8], which who did not employ quality control, and found over 90% to have at least one inconsistent violation. In our sample, that proportion for SA perspectives was 27%. Second, this study used a sample of students, whereas valuation studies use samples representative of the general adult population [11, 21, 28, 40]. Hence, the small differences observed between child and adult perspectives may be explained by the fact that our sample consisted of respondents who are still transitioning into adulthood. While students may remember their recent childhood better than older adults, making it easier for them to value health states for a child perspective, they will likely not (yet) have any children of their own, which may be relevant for EQ-5D-Y-3L valuation. It is also well known that some of the demographics in which students differ from the general public may affect health state valuation, such as age and education level [8, 25, 47]. Furthermore, even though the student sample included a students of many nationalities, there is evidence of Dutch EQ-5D valuation having different characteristics [33]. Hence, it is recommended that future work considers to replicate our approach in general public samples. Third, the bisection choice procedure implemented in this study differs from the elicitation procedure used or recommended by others [21, 37]. Although this change facilitated self-completion, it is well known that the elicitation procedure can influence TTO results [3]. Fourth, although we aimed for both health state blocks to be assigned randomly, the final distribution was imbalanced suggesting randomisation error could have taken place. Finally, in order to increase power, we opted for a within-subjects approach, in which the order of each perspective was randomized. Although such an approach will help identify within-subjects effects and differences, it may also be sensitive to order effects and perhaps anchoring [45]. That is, individuals may have anchored on their initial answer for a single perspective and adjusted their answers for subsequent perspectives insufficiently. Although randomizing the order ensures this does not systematically bias our results on a sample level, anchoring could explain why the differences in outcomes between perspectives appear smaller than in some of the findings published in earlier work. Future research could test this hypothesis using the four perspectives we used in a between-subjects experiment.

To conclude, our study showed that the use of different perspectives will likely yield (at least) small, but systematic differences in the outcomes, precision, and quality of valuation of EQ-5D-Y-3L health states. Our exploration of the causes of these effects suggested that TTO utilities are affected in upward direction when one is asked to decide for others (rather than for oneself). Deciding for children (rather than adults) can affect EQ-5D-Y-3L valuation with both VAS and TTO, but the direction of this effect was ambiguous and differed between respondents and health states in our study. If these results generalise to the samples used in EQ-5D-Y-3L valuation, the small observed differences may have consequences for estimation of QALY gains [26]. Nonetheless, the large heterogeneity between respondents and states we found suggests that the search for the empirical and normative implications of perspective used in EQ-5D-Y-3L is far from over.

## Supplementary Information

Below is the link to the electronic supplementary material.

Supplementary file1 (DOCX 189 kb)

## Footnotes

1 Note that the exact instruction included in the protocol is: ‘Considering your views about a 10-year-old child, what do you prefer?’(p. 658, [35].

2 Power-analysis based on a paired t-test with Cohen’s *d* = 0.2, *α* = 0.05, *β *= 0.8, i.e., using the parametrization proposed by Cohen (1988).

3 We opted for a pre-recorded video introduction to the valuation tasks to comply with the local COVID-19 regulations.
