# Supplementary material for: Self vs. other, child vs. adult. An experimental comparison of valuation perspectives for valuation of EQ-5D-Y-3L health states
Source: Eur J Health Econ. 2021 Oct 6;22(9):1507–18. doi: 10.1007/s10198-021-01377-y (PMC8492455; doi:10.1007/s10198-021-01377-y)
Supplement: Supplementary file 1 — Supplementary file1 (DOCX 189 kb) [file 10198_2021_1377_MOESM1_ESM.docx]

**Online supplementary file for publication**

Self vs. other, child vs. adult. An experimental comparison of valuationperspectives for valuation of EQ-5D-Y-3L health states

S. A. Lipman · V. T. Reckers-Droog · M. Karimi · M. Jakubczyk · A. E. Attema

Corresponding author: lipman@eshpm.eur.nl

**Appendix A. Instructions and example tasks**

The video with instructions for this project as well as a demonstration of each task is (anonymously) hosted here: <https://vimeo.com/500429242>. This video also shows examples of how the tasks were operationalised.

**Appendix B. Additional results, tables and figures.**

This appendix contains additional results, that may help interpret the findings of our work. In particular, the following additional analyses are reported.

- 1. Mean and standard deviations (SD) per state and perspective. Table B1 reports mean and SD for all states included in this experiment, split by block, method and perspective.
  2. Mixed effects regression models per health state. We explored the effect of perspectives per health state by running the linear mixed-effects regression models for each state. As for the models reported in Table 3 in the main text, models were specified with subject random effects with the following fixed effects: 1) Δ(A-C), 2) Δ(S-O), and 3) Δ(A-C) x Δ(S-O). Results are reported in Table B2
  3. Mixed effects regression for within-subjects variance of utilities: We also analysed the variance of valuations of health states per respondent and perspective (i.e. within-subjects) to assess whether, for instance, for one perspective the valuations tend to be contracted towards the middle of the scale, i.e. mild states are assigned a lower and severe states a greater utility. As such, these analyses determine if differences exist in the spread of valuations across the severity scale. The contribution of Δ(A-C) and Δ(S-O) was determined by performing mixed effects regression models with subject random effects with the following fixed effects: 1) Δ(A-C), 2) Δ(S-O), and 3) Δ(A-C) x Δ(S-O). Results are reported in Table B2 as well.
  4. Parsimonious mixed effects regression models. The models reported in Table 3 included interaction terms that were not significant. In Table B3 we show the results of these regression analyses without interaction terms, i.e. these tables allow the reader to see the results of parsimonious model specifications.
  5. Distribution of TTO and VAS valuations. Figures B1 to B3 depict the distributions of VAS scores for each of the 8 health states included in this experiment, and Figures B4 to B6 depict the distribution of TTO utilities for each of the 8 health states. Finally, the overall distribution of valuations for both methods is found in Figure B7.
  6. Within-subjects categorisation of patterns per health state: Figure 1 in the main paper shows the correlation of difference scores calculated between perspectives. In Table B4 and B5, the sign of these difference scores is interpreted per health state. These tables also include per health state Pearson correlation coefficients.
  7. Bayesian model specification: The code used for specifying the Bayesian model to test for differences in variances is included in Box BI for VAS and Box BII for TTO.

**Table B1.** Mean (SD) VAS scores and TTO utilities elicited by state, method and perspective

| VAS scores | | | | |
| --- | --- | --- | --- | --- |
| **Block 1 (n=88 )** | **Self-adult** | **Other-adult** | **Self-child** | **Other-child** |
| 11121 | 85.24 (10.00) | 83.52 (10.18) | 82.22 (12.21) | 81.3 (11.51) |
| 32211 | 63.2 (22.86) | 62.64 (21.02) | 60.84 (24.11) | 62.86 (23.32) |
| 33323 | 27.92 (24.29) | 28.48 (23.29) | 29.78 (26.71) | 30.35 (27.09) |
| **Block 2 (n=117 )** | **Self-adult** | **Other-adult** | **Self-child** | **Other-child** |
| 11112 | 86.97 (9.24) | 86.84 (8.52) | 85.43 (11.35) | 85.61 (11.15) |
| 11312 | 68.32 (19.96) | 67.35 (18.06) | 68.97 (23.27) | 70.86 (20.73) |
| 13311 | 55.17 (21.21) | 56.29 (19.97) | 58.8 (23.65) | 60.43 (22.09) |
| **Blocks 1 & 2 (n=205)** | **Self-adult** | **Other-adult** | **Self-child** | **Other-child** |
| 22222 | 50.34 (21.68) | 51.39 (20.27) | 50.38 (23.22) | 49.61 (22.69 |
| 33333 | 18.9 (25.53) | 20.91 (25.77) | 21.19 (28.99) | 19.84 (27.03) |
|  |  |  |  |  |
| TTO utilities | | | | |
| **Block 1 (n=88 )** | **Self-adult** | **Other-adult** | **Self-child** | **Other-child** |
| 11121 | 0.88 (0.17) | 0.85 (0.16) | 0.87 (0.23) | 0.86 (0.12) |
| 32211 | 0.65 (0.31) | 0.66 (0.3) | 0.66 (0.34) | 0.66 (0.31) |
| 33323 | 0.13 (0.61) | 0.2 (0.55) | 0.21 (0.57) | 0.26 (0.54) |
| **Block 2 (n=117 )** | **Self-adult** | **Other-adult** | **Self-child** | **Other-child** |
| 11112 | 0.87 (0.11) | 0.87 (0.1) | 0.85 (0.14) | 0.87 (0.12) |
| 11312 | 0.64 (0.29) | 0.65 (0.23) | 0.62 (0.34) | 0.67 (0.27) |
| 13311 | 0.61 (0.36) | 0.65 (0.30) | 0.66 (0.34) | 0.69 (0.28) |
| **Blocks 1 & 2 (n=205)** | **Self-adult** | **Other-adult** | **Self-child** | **Other-child** |
| 22222 | 0.61 (0.34) | 0.65 (0.26) | 0.61 (0.33) | 0.61 (0.35) |
| 33333 | -0.16 (0.60) | -0.12 (0.57) | -0.10 (0.60) | -0.12 (0.59) |

**Table B2.** Mixed effects regression coefficients (standard errors in brackets) per health state

|  | **VAS** |  |  |  |  | **TTO** |  |  |  |
| --- | --- | --- | --- | --- | --- | --- | --- | --- | --- |
|  | **(Intercept)** | **Δ(A-C): C** | **Δ(S-O): O** | **Δ(S-O) x Δ(A-C): OC** |  | **(Intercept)** | **Δ(A-C): C** | **Δ(S-O): O** | **Δ(S-O) x Δ(A-C): OC** |
| **11121** | 85.24 (1.17)*** | -3.02 (0.84)*** | -1.72 (0.84)* | 0.80  (1.19) |  | 0.88 (0.02)*** | -0.01  (0.01) | -0.02 (0.01)+ | 0.02 (0.02) |
| **32211** | 63.20 (2.44)*** | -2.36 (1.35)+ | -0.57  (1.35) | 2.59  (1.91) |  | 0.65 (0.03)*** | 0.00  (0.02) | 0.00  (0.02) | 0.00  (0.03) |
| **33323** | 27.92 (2.71)*** | 1.86  (1.15) | 0.56  (1.15) | 0.01  (1.62) |  | 0.13 (0.06)* | 0.08 (0.05)+ | 0.07  (0.05) | -0.02 (0.07) |
| **11112** | 86.97 (0.94)*** | -1.55 (0.67)* | -0.14  (0.67) | 0.32  (0.94) |  | 0.87 (0.01)*** | -0.02 (0.01)* | 0.00 (0.01) | 0.01  (0.01) |
| **11312** | 68.32 (1.90)*** | 0.65  (1.20) | -0.97  (1.20) | 2.86 (1.69)+ |  | 0.64 (0.03)*** | -0.02 (0.02) | 0.01  (0.02) | 0.04  (0.03) |
| **13311** | 55.17 (2.01)*** | 3.63 (1.20)** | 1.12  (1.20) | 0.50  (1.70) |  | 0.61 (0.03)*** | 0.05 (0.03)+ | 0.04 (0.03)+ | -0.01 (0.04) |
| **22222** | 50.34 (1.54)*** | 0.03  (0.81) | 1.04  (0.81) | -1.80 (1.15) |  | 0.61 (0.02)*** | 0.00  (0.02) | 0.05 (0.02)** | -0.05 (0.02)+ |
| **33333** | 18.9 (1.88)*** | 2.29 (0.75)** | 2.00  (0.75)** | -3.35 (1.07)** |  | -0.16 (0.04)*** | 0.05 (0.03)* | 0.03 (0.03) | -0.05 (0.04) |
|  | Within-subjects variance | | | | | | | | |
| **Variance** | 966.96  (32.12)*** | -30.14  (16.66)+ | -69.21  (16.66)*** | 57.94  (23.56)** |  | 0.28  (0.02)*** | -0.03  (0.01)* | -0.02 (0.01)+ | 0.03  (0.01)+ |

**Note:** ***, **, * and + indicate (marginal) significance at *p* < 0.001, p < 0.01, p < 0.05 and p<0.10, respectively.

The results for these mixed effects regression models per health state suggested that for VAS, deciding for children was associated with statistically significantly lower valuations for state 11121, 11112, 32211 (marginally significant) as compared to deciding for adults. The opposite held for states 13311 and 33333 as these states were assigned statistically significantly higher values for children. Similar findings were observed for deciding for others for VAS, with a significant lower score for state 11121 and higher score for state 33333. The difference was (marginally) significant for state 11312 and 33333, where deciding for other children was associated with a higher score for state 11312 and lower score for state 33333. Overall, these results suggest that if differences were observed between perspectives for VAS, the direction of these effects depends on the severity of the health state selected. For TTO, (marginally) significant effects of deciding for children (rather than adults) were observed in both directions. TTO utilities were, on average, lower for children for state 11112, and higher for children for state 13311, 33323 and 33333. Deciding for others (rather than for oneself) was associated with (marginally) significantly higher TTO utilities for states 11121, 13311, and 33333. The interaction effect between Δ(S-O) and Δ(A-C) was only (marginally) significant for state 22222, suggesting few systematic effects of deciding for other children for TTO.

Our analysis of within-subjects variance is reported in Table B2. The results of these analyses indicate that the perspective used affected the spread of valuations, with the directions of these effects being similar for VAS and TTO. Deciding for others was associated with (marginally) significantly more condensed valuations, and hence less variance than deciding for oneself. This was also observed when deciding for children for both methods as compared to deciding for adults. However, the (marginally) significant positive interaction term between Δ(S-O) and Δ(A-C) suggests that deciding for other children was associated with a larger spread, and hence variance of valuations.


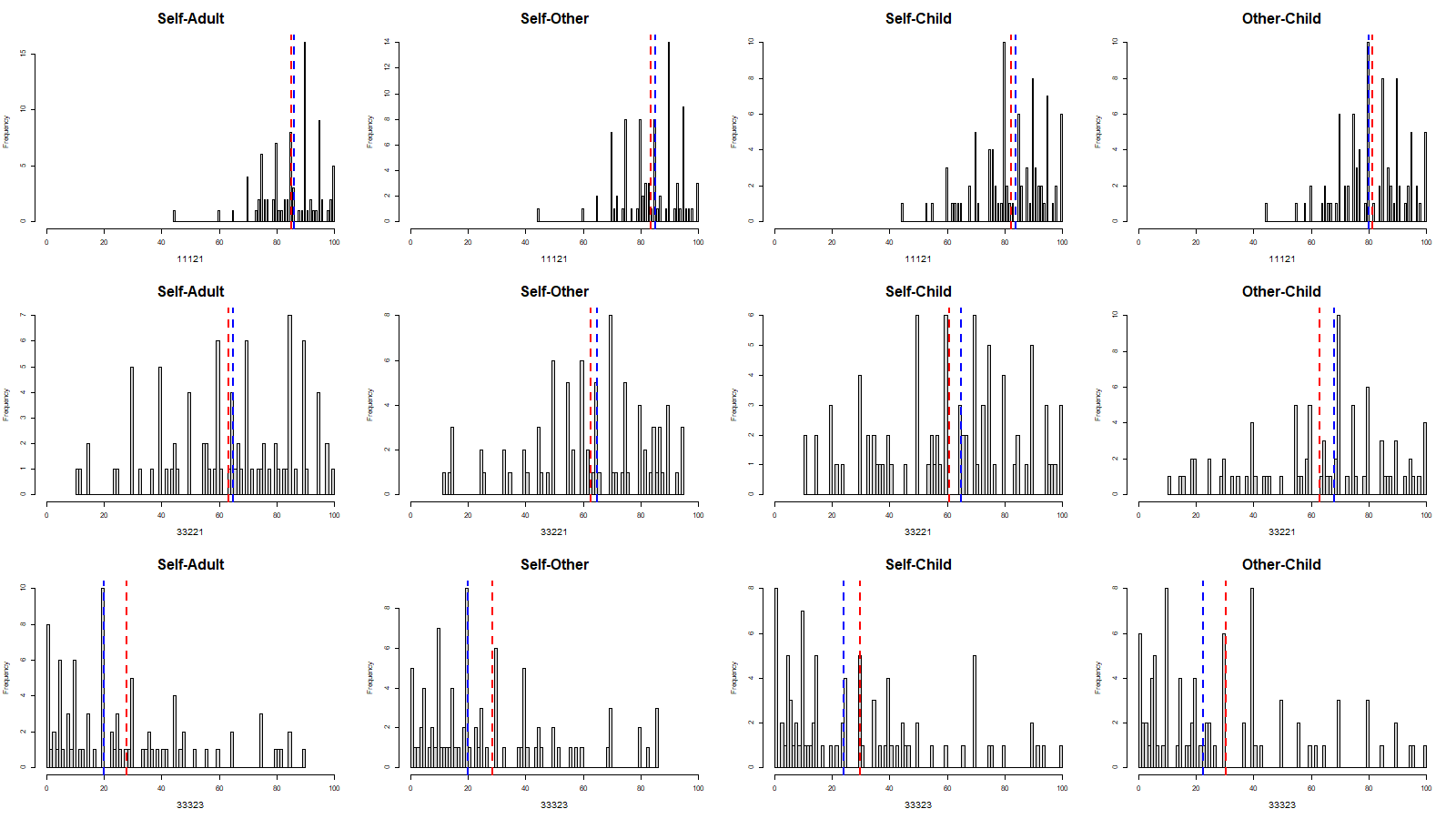


*Figure B1:* Distribution of VAS scores for states 11121, 32211, and 33323 (*n= 88)* per perspective, with means (in red) and medians (in blue)

*
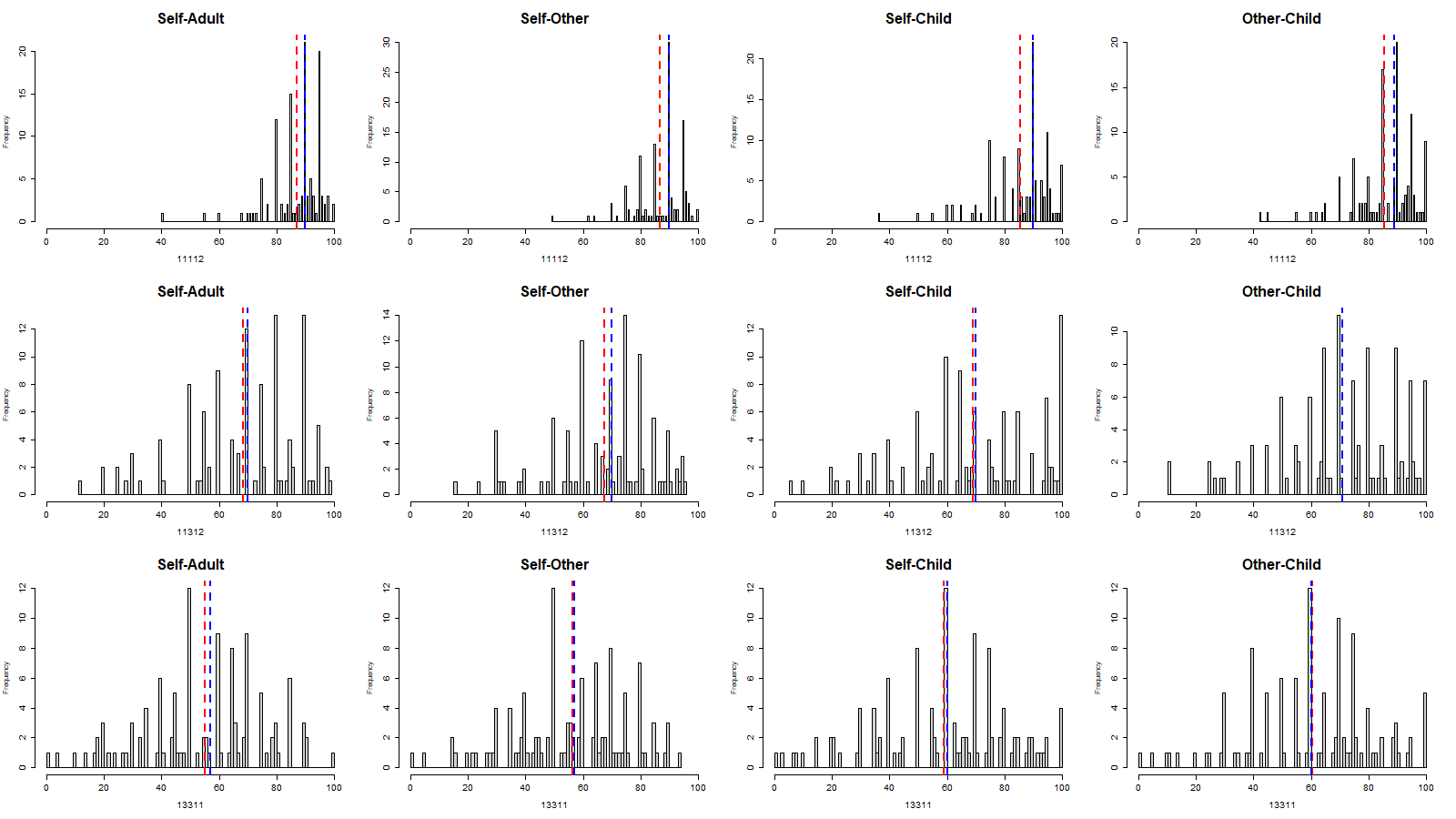
*

*Figure B2:* Distribution of VAS scores for states 11112, 11312, and 13311 (*n= 117)* per perspective, with means (in red) and medians (in blue)

*
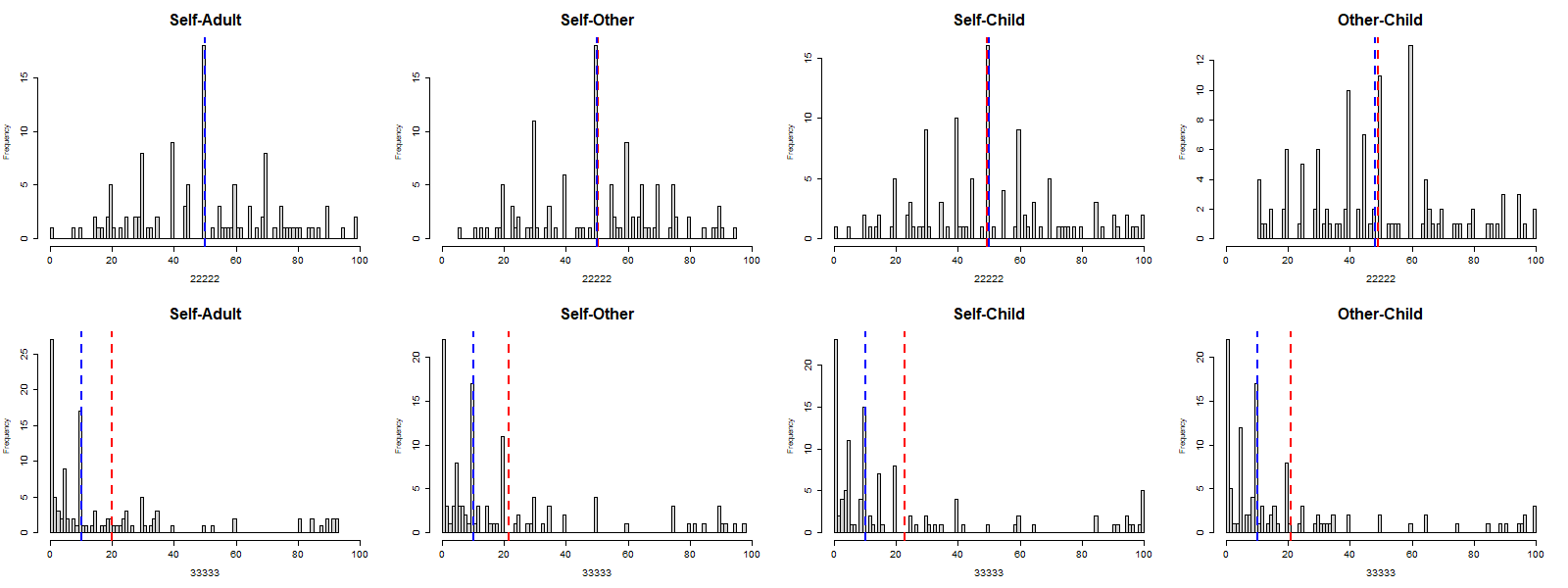
Figure B3:* Distribution of VAS scores for states 22222, and 33333 (*n= 205)* per perspective, with means (in red) and medians (in blue)

*
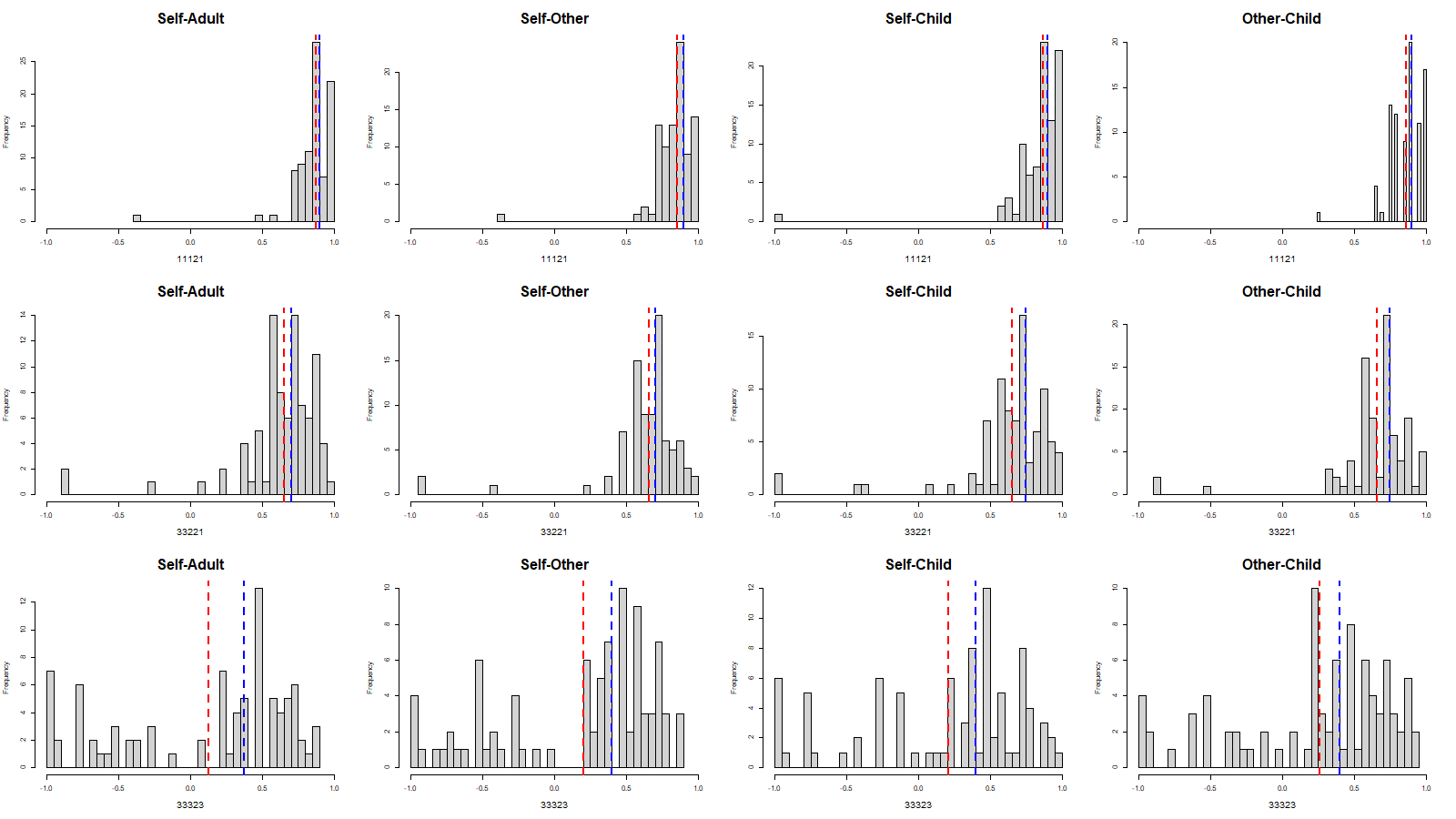
Figure B4:* Distribution of TTO utilities for states 11121, 32211, and 33323 (*n= 88)* per perspective, with means (in red) and medians (in blue)

*
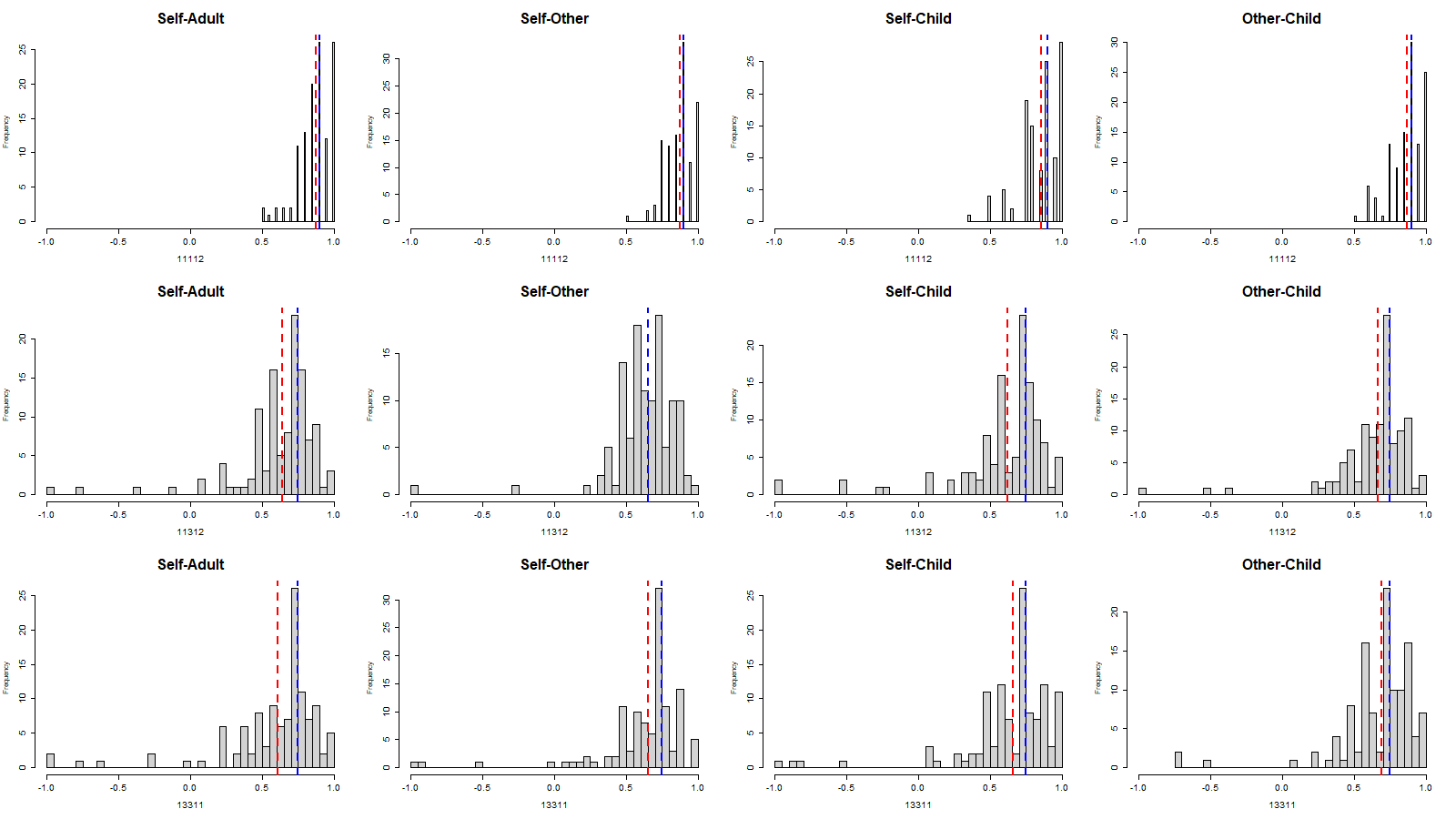
Figure B5:* Distribution of TTO utilities for states 11112, 11312, and 13311 (*n= 117)* per perspective, with means (in red) and medians (in blue)

*
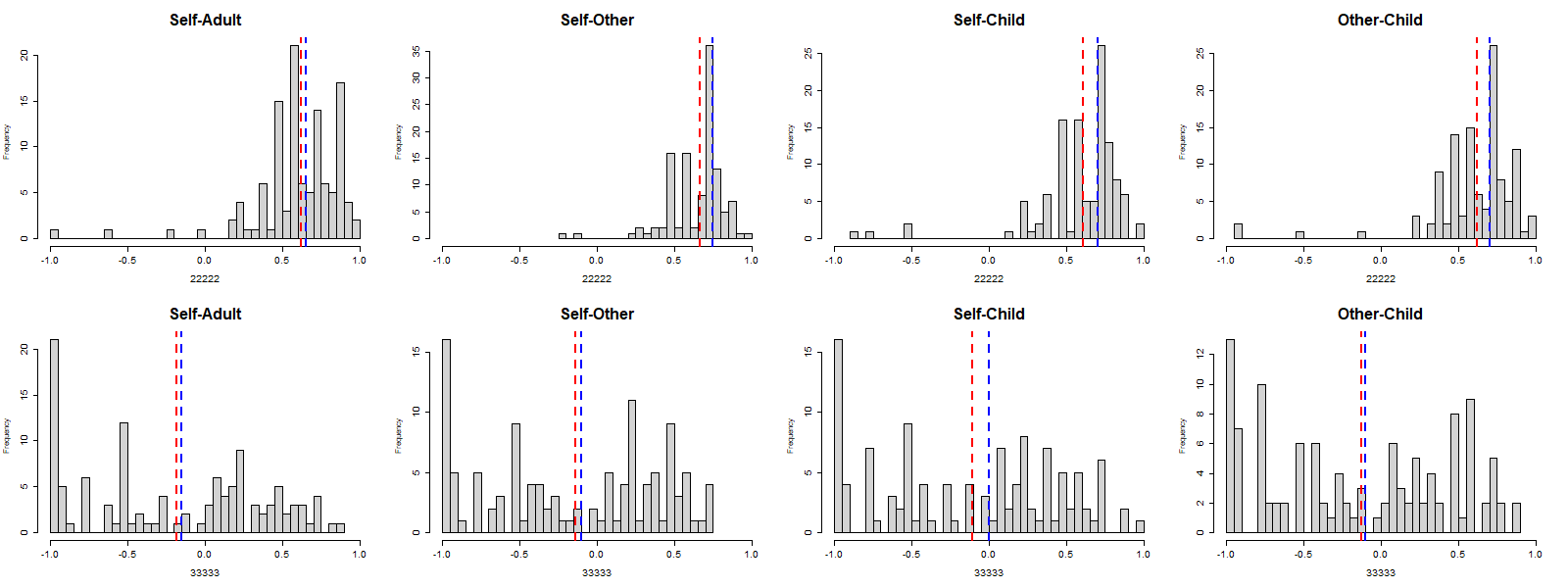
Figure B6:* Distribution of TTO utilities for states 22222, and 33333 (*n= 205)* per perspective, with means (in red) and medians (in blue)


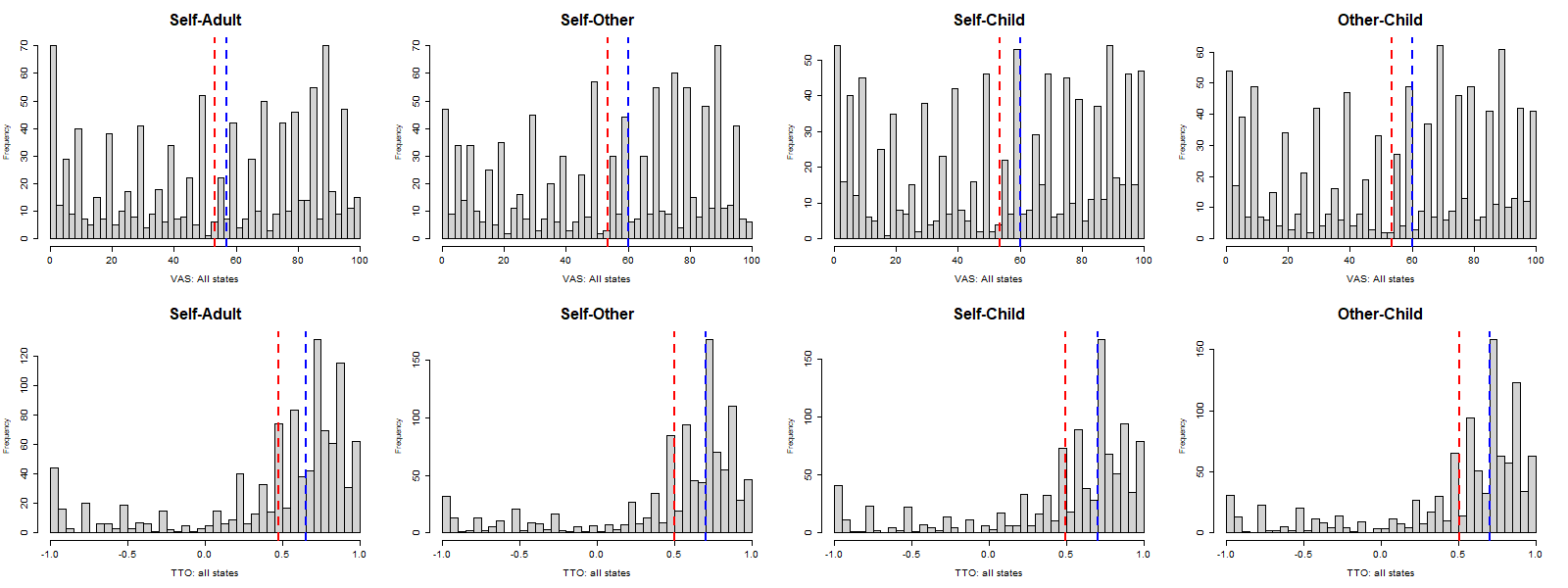


Figure B7: Overall distributions by perspective and method with mean (in red) and median (in blue) valuations

**Table B1.** Results for models 1 to 4 (as reported in Table 3) without interaction effects.

| **Model** | **1** | **2** | **3** | **4** |
| --- | --- | --- | --- | --- |
| Method | VAS | TTO | VAS | TTO |
|  |  |  |  |  |
| Intercept | 85.82  (1.32) *** | 0.84  (0.02) *** | 81.97  (1.10)*** | 0.94  (0.02) *** |
| **Fixed effects** |  |  |  |  |
| Δ(A-C): C | 0.31  (0.53) | 0.01  (0.01) | 0.31  (0.53) | 0.01  (0.01) |
| Δ(S-O): O | 0.30  (0.53) | 0.02  (0.01)* | 0.30  (0.53) | 0.02  (0.01)+ |
| LSS |  |  | -7.02  (0.08)*** | -0.11  (0.003)*** |
| HS: 11121 | -2.95  (1.42)* | 0.00  (0.03) |  |  |
| HS: 11312 | -17.33  (1.1)*** | -0.22  (0.02)*** |  |  |
| HS: 13311 | -28.54  (1.1)*** | -0.22  (0.02)*** |  |  |
| HS: 22222 | -35.70  (1.03)*** | -0.25  (0.03)*** |  |  |
| HS: 32211 | -23.63  (1.42)*** | -0.21  (0.03)*** |  |  |
| HS: 33323 | -56.88  (1.42)*** | -0.67  (0.03) *** |  |  |
| HS: 33333 | -65.92  (1.03)*** | -0.99  (0.02)*** |  |  |

**Note:** ***, **, * and + indicate (marginal) significance at p<0.001, p<0.01, p<0.05 and p<0.10, respectively.

The results of these regressions shows that the results reported in Table 3 are robust to exclusion of (non-significant) interaction effects.

**Table B4:** The effect of deciding for children rather than adults: response patterns when comparing SC and OC perspectives with SA and OA perspectives.

|  | VAS |  |  |  |  |  |  |
| --- | --- | --- | --- | --- | --- | --- | --- |
|  | </< | =/< | =/= | >/< | >/= | >/> |  |
| **Block 1** |  |  |  |  |  | *Pearson’s r* | |
| 11121 | 21 | 4 | 6 | 8 | 9 | 40 | 0.71*** |
| 32211 | 28 | 3 | 3 | 18 | 8 | 28 | 0.43*** |
| 33323 | 22 | 10 | 10 | 12 | 6 | 28 | 0.77*** |
| **Block 2** |  |  |  |  |  |  |  |
| 11112 | 36 | 10 | 8 | 13 | 5 | 45 | 0.73*** |
| 11312 | 48 | 6 | 6 | 19 | 5 | 33 | 0.72*** |
| 13311 | 48 | 8 | 7 | 19 | 7 | 28 | 0.68*** |
| **Blocks 1 & 2 (n=205)** |  |  |  |  |  |  |  |
| 22222 | 56 | 13 | 14 | 40 | 18 | 64 | 0.53*** |
| 33333 | 41 | 18 | 40 | 34 | 25 | 47 | -0.04 |
| ***Total*** | ***300*** | ***72*** | ***94*** | ***163*** | ***83*** | ***313*** | ***0.51****** |
|  | TTO |  |  |  |  |  |  |
|  | </< | =/< | =/= | >/< | >/= | >/> |  |
| **Block 1** |  |  |  |  |  |  |  |
| 11121 | 9 | 16 | 15 | 19 | 20 | 9 | -0.35*** |
| 32211 | 24 | 16 | 11 | 13 | 11 | 13 | 0.22* |
| 33323 | 27 | 9 | 7 | 18 | 11 | 16 | 0.55*** |
| **Block 2** |  |  |  |  |  |  |  |
| 11112 | 17 | 17 | 27 | 18 | 12 | 26 | 0.14 |
| 11312 | 23 | 26 | 10 | 21 | 16 | 21 | 0.64*** |
| 13311 | 38 | 18 | 9 | 17 | 17 | 18 | 0.34*** |
| **Blocks 1 & 2 (n=205)** |  |  |  |  |  |  |  |
| 22222 | 26 | 43 | 24 | 44 | 29 | 39 | 0.22** |
| 33333 | 56 | 28 | 28 | 27 | 29 | 37 | 0.33*** |
| ***Total*** | ***220*** | ***173*** | ***131*** | ***177*** | ***145*** | ***179*** | ***0.38****** |

**Note:** >,=,< indicate that the elicited valuation with the child perspective was higher than, equal to, or lower than the valuation with the adult perspective. For example, </< indicates respondents for whom SC (OC) valuation was higher than SA (OA). *, ** and *** indicate that the Pearson correlation coefficient was significant at p<0.05, p<0.01 and p<0.001 respectively.

**Table B5:** The effect of deciding for self rather than others: response patterns when comparing SA and SC perspectives with OA and OC perspectives.

|  | VAS |  |  |  |  |  |  |
| --- | --- | --- | --- | --- | --- | --- | --- |
|  | </< | =/< | =/= | >/< | >/= | >/> |  |
| **Block 1 (n=88)** |  |  |  |  |  | *Pearson’s r* | |
| 11121 | 6 | 12 | 12 | 19 | 14 | 25 | 0.35** |
| 32211 | 16 | 19 | 7 | 20 | 10 | 16 | -0.10 |
| 33323 | 19 | 17 | 21 | 18 | 5 | 8 | 0.15 |
| **Block 2 (n=117)** |  |  |  |  |  |  |  |
| 11112 | 21 | 13 | 28 | 21 | 22 | 12 | 0.28 |
| 11312 | 19 | 19 | 18 | 23 | 24 | 14 | 0.13 |
| 13311 | 21 | 29 | 15 | 20 | 19 | 13 | 0.005 |
| **Blocks 1 & 2 (n=205)** |  |  |  |  |  |  |  |
| 22222 | 27 | 44 | 26 | 52 | 24 | 32 | 0.18* |
| 33333 | 31 | 33 | 53 | 33 | 33 | 22 | -0.20** |
| ***Total*** | ***160*** | ***186*** | ***180*** | ***206*** | ***151*** | ***142*** | ***0.001*** |
|  | TTO |  |  |  |  |  |  |
|  | </< | =/< | =/= | >/< | >/= | >/> |  |
| **Block 1** |  |  |  |  |  |  |  |
| 11121 | 4 | 17 | 15 | 14 | 23 | 15 | 0.09 |
| 32211 | 14 | 16 | 13 | 13 | 14 | 18 | 0.08 |
| 33323 | 13 | 25 | 10 | 21 | 9 | 10 | 0.17 |
| **Block 2** |  |  |  |  |  |  |  |
| 11112 | 10 | 20 | 42 | 19 | 18 | 8 | -0.30*** |
| 11312 | 15 | 32 | 13 | 18 | 26 | 13 | 0.22* |
| 13311 | 17 | 28 | 20 | 18 | 26 | 8 | 0.09 |
| **Blocks 1 & 2 (n=205)** |  |  |  |  |  |  |  |
| 22222 | 29 | 53 | 32 | 40 | 30 | 21 | 0.10 |
| 33333 | 42 | 35 | 31 | 37 | 35 | 25 | 0.14 |
| ***Total*** | ***144*** | ***226*** | ***176*** | ***180*** | ***181*** | ***118*** | ***0.13****** |

**Note:** >,=,< indicate that the elicited valuation with the self-perspective was higher than, equal to or lower than the valuation with the other perspective. For example, </< indicates respondents for whom SA (SC) valuation was higher than OA (SC).

*, ** and *** indicate that the Pearson correlation coefficient was significant at p<0.05, p<0.01 and p<0.001 respectively.

**Box BI: JAGS model VAS data**

model

{

for (i in 1:nResp){

disuScale[i] ~ dnorm(0,tauDisuSc)

sdScale[i] ~ dnorm(0,tauVarSc)

}

for (i in 1:8){

stateDisu[i] ~ dunif(0,100)

}

tauDisuSc ~ dgamma(.0001,.0001)

tauVarSc ~ dgamma(.0001,.0001)

ifOthersd ~ dunif(0,10)

ifAdultsd ~ dunif(0,10)

stateSDconst ~ dunif(0,25)

for (i in 1 : nTTO){

avgDisu[i] <- stateDisu[state[i]] * (1+disuScale[resp[i]])

avgSD[i] <- stateDisu[state[i]]*stateSDconst * pow(ifOthersd,equals(perspOwn[i],0)) * pow(ifAdultsd,equals(perspAdult[i],0))

disu[i] ~ dnorm(avgDisu[i],pow(avgSD[i],-2))

}

# data # nResp, resp, nTTO, state, perspOwn, perspAdult, perspOC

# data # disu

# monitor # stateDisu, ifOthersd, ifAdultsd

# monitor # tauDisuSc, tauVarSc, stateSDconst

}

**Box BII: JAGS model – TTO data**

model

{

for (i in 1:nResp){

disuScale[i] ~ dnorm(0,tauDisuSc)

sdScale[i] ~ dnorm(0,tauVarSc)

}

for (i in 1:8){

stateDisu[i] ~ dunif(0,2)

}

tauDisuSc ~ dgamma(.0001,.0001)

tauVarSc ~ dgamma(.0001,.0001)

ifOthersd ~ dunif(0,10)

ifAdultsd ~ dunif(0,10)

stateSDconst ~ dunif(0,1)

for (i in 1 : nTTO){

avgDisu[i] <- stateDisu[state[i]] * (1+disuScale[resp[i]])

avgSD[i] <- stateDisu[state[i]]*stateSDconst * pow(ifOthersd,equals(perspOwn[i],0)) * pow(ifAdultsd,equals(perspAdult[i],0))

disu[i] ~ dnorm(avgDisu[i],pow(avgSD[i],-2))

}

# data # nResp, resp, nTTO, state, perspOwn, perspAdult

# data # disu

# monitor # stateDisu, ifOthersd,ifAdultsd

# monitor # tauDisuSc, tauVarSc, stateSDconst

}
